# Comparison of 3 diagnostic platforms for identification of bacteria and yeast from positive blood culture bottles

**DOI:** 10.1016/j.diagmicrobio.2023.116018

**Published:** 2023-07-07

**Authors:** Richard D. Smith, J. Kristie Johnson, Robert K. Ernst

**Affiliations:** a Department of Pathology, School of Medicine, University of Maryland, Baltimore, MD, USA; b Department of Microbial Pathogenesis, School of Dentistry, University of Maryland, Baltimore, MD, USA

**Keywords:** Blood culture, MALDI-TOF MS, Clinical microbiology

## Abstract

Managing bloodstream infections requires fast and accurate diagnostics. Culture-based diagnostic methods for identification from positive blood culture require 24-hour subculture, potentially delaying time to appropriate therapy. Positive blood cultures were collected (n = 301) from September 2021 to August 2022 at the University of Maryland Medical Center. Platforms compared were BioFire^®^ BCID2, Sepsityper^®^, and short-term culture. For monomicrobial cultures, FilmArray^®^ BCID2 identified 88.3% (241/273) of pathogens. Rapid Sepsityper^®^ identified 76.9% (210/273) of pathogens. Sepsityper^®^ extraction identified 82.4% (225/273) of pathogens. Short-term culture identified 83.5% (228/273) of pathogens. For polymicrobial cultures, Sepsityper^®^, short-term culture, and BioFire^®^ BCID2 had complete identifications at 10.7% (3/28), 0%, and 92.9% (26/28), respectively. Time-to-results for Rapid Sepsityper^®^, Sepsityper^®^ extraction, BioFire^®^ BCID2, and Short-term culture were 35, 52, 65, and 306 minutes, respectively. Performance of these platforms can reduce time-to-results and may help effectively treat bloodstream infections faster. Accuracy, time-to-result, and hands-on time are important factors when evaluation diagnostic platforms.

## 1. Introduction

Bloodstream infections (BSIs) are defined as a systemic infection resulting from the presence of viable microorganisms in the blood [1,2]. BSIs are associated with high rates of morbidity and mortality, are a leading cause of death, globally, and a common cause of the life-threatening condition, sepsis [3,4]. BSIs are especially problematic in intensive care units (ICU) where an estimated 5% of admitted patients will be afflicted with a BSI with a case fatality rate of 35% to 50% [5]. To best manage BSIs, early treatment with effective antimicrobial therapy is critical. Kumar et al. [6] demonstrated that among ICU patients, from onset of sepsis-associated hypotension, delays in administration of effective antibiotics were associated with higher rates of mortality. Moreover, for each hour effective antimicrobials were delayed, survival decreased by approximately 7.6%. One method to reduce time to effective antimicrobial therapy and reduce morbidity and mortality associated with BSIs is implementation of fast and accurate diagnostic testing.

Currently, blood culture remains the gold standard diagnostic test for BSIs [7]. After patient examination indicate signs and symptoms of a potential BSI such as fever, chills, hypotension, change in mental status, or tachychardia, blood cultures are ordered [8]. Typically, a minimum of 2 sets blood cultures, including 1 aerobic and 1 anaerobic blood culture bottle, are collected from different body sites. Once positive, standard identification methods, such as biochemical-based methods or Matrix Assisted Laser Desorption/Ionization-Time of Flight Mass Spectrometry (MALDI-TOF MS), require subculture to solid media and incubation for 24 to 48 hours [7]. Thus, this requirement to subculture further delays results and time to appropriate therapy.

MALDI-TOF MS has emerged as a crucial component in the clinical microbiology setting due to its easy-to-use, accurate, and high-throughput nature [9]. Furthermore, the 2 FDA-cleared protein-based MALDI-TOF MS platforms, MALDI Biotyper^™^ (Bruker Daltonics, Billerica, MA) and Vitek MS (bioMérieux, Inc., Durham, NC), have extensive organism libraries [10]. Despite these strengths, a major limitation of traditional MALDI-TOF MS methods is the need to culture for 24 hours on solid media to obtain an isolated colony, delaying time to identification. However, studies have shown MALDI-TOF MS can identify microorganisms as soon as growth is observed [11]. This method is known as the short-term culture or scant growth method. To further reduce time to identification via MALDI-TOF MS, direct from blood culture sample preparation assays for MALDI-TOF MS have been developed [12]. The MBT Sepsityper^®^ kit (US-IVD) (Bruker Daltonics, Billerica, MA), the first direct from blood MALDI-TOF MS assay to be cleared by the United States Food and Drug Administration (FDA), has previously shown to produce identification in approximately 30 minutes from blood culture positivity [13].

Furthermore, rapid, direct from positive blood culture, molecular assays such as BioFire^®^ FilmArray^®^ (bioMérieux Inc., Durham, NC) and Verigene^®^ (Luminex Corp, Austin, TX), have emerged in the clinical microbiology setting [14]. These assays are known to have quick turnaround times, minimal hands-on time, and high sensitivities [15]. However, these methods can be expensive and contain limited organism panels.

Previous studies have evaluated Sepsityper^®^ prior to FDA-clearance; however, few studies evaluating Sepsityper^®^ since FDA clearance have been published. Moreover, literature comparing the FDA cleared Sepsityper^®^ to rapid molecular assays, such as BioFire^®^ FilmArray^®^, are lacking. In this study, we evaluated and compared 3 diagnostic platforms that can reduce time to identification compared to traditional culture methods: BioFire^®^ BCID2, Sepsityper^®^, and the short-term culture method.

## 2. Materials and methods

*Collection of positive blood cultures* positive blood cultures (n = 301) were collected prospectively at the University of Maryland Medical Center (UMMC) from September 2021 to August 2022 (IRB HP-76072). Once identified as positive by BacT/alert^®^ 3D (bioMérieux Inc, Durham, NC), blood cultures were Gram-stained and stored at room temperature. All positive blood cultures collected in this study were processed and analyzed within 12 hours of positivity. Only the first positive blood culture for each patient was collected.

*Biofire*^®^ *FilmArray*^®^ *BCID2* the BioFire^®^ BCID2^®^ (bioMérieux Inc,Durham, NC) panel is routinely performed on the first positive blood culture per each patient by a UMMC clinical laboratory staff member as per manufacturer instructions.

*Rapid Sepsityper*^®^ positive blood cultures were inverted to mix and 1 mL of the positive blood culture was transferred to 1.5 mL tubes included in the Sepsityper^®^ kit (Bruker Daltonics, Billerica, MA) following manufacturer’s instructions [16]. Next, 200 *μ*L of lysis buffer was added and tubes were inverted twice then vortexed for 10 to 15 seconds. Samples were then centrifuged for 2 minutes at 13,000 rpm. After centrifugation, supernatant was removed, and the bacterial pellet was resuspended in 1 mL of washing buffer. The resuspended samples were centrifuged for 1 minute at 13,000 rpm. Supernatant was discarded and, using a rounded toothpick, the pellet is smeared onto 2 spots on the target. Rapid Sepsityper^®^ includes 2 extraction methods, Direct Transfer (DT) and extended Direct Transfer (eDT). On 1 of the spots, no further extraction was performed (DT). On the other spot, 1 *μ*L of 70% formic acid was spotted overtop the sample (eDT). After the formic acid dries, 1 *μ*L of matrix (HCCA) was added to each spot. Samples were analyzed via MALDI Biotyper^®^ Sirius CA system (US-IVD) using the Sepsityper^®^ database.

*Sepsityper*^®^ extraction according to manufacturer instructions, the extraction protocol is conducted when no identification or a low confidence identification is observed after Rapid Sepsityper^®^. Sepsityper^®^ extraction protocol was completed after the Rapid Sepsityper^®^ protocol was completed for all collected samples. With the remaining pellet from the Rapid Sepsityper^®^ extraction, 300 *μ*L of sterile water was added and the pellet was resuspended. Next, 900 *μ*L of ethanol was added to the resuspended pellet. The sample was then centrifuged for 2 minutes at 13,000 rpm. After centrifugation, supernatant was aspirated, and centrifugation was repeated. Remaining supernatant was removed and the pellet was air dried for a minimum of 5 minutes. Then, equal amounts, typically 20 *μ*L, of 70% formic acid and acetonitrile were added and vortexed for approximately 10 seconds. Lastly, 1 *μ*L of supernatant was spotted, in duplicate, on the target, allowed to dry, then overlayed with 1 *μ*L of HCCA matrix. Samples after extraction were analyzed via MALDI Biotyper^®^ Sirius CA system (US-IVD) using the Sepsityper^®^ database.

*Short-term culture method* in theory, MALDI-TOF MS allows for identification of pathogens as soon as growth is observed in culture [17]. Despite this, standard MALDI-TOF MS analysis in clinical laboratories is conducted after 18 to 24 hours [18]. To conduct the short-term culture method, 10 *μ*L of the positive blood culture was subcultured on tryptic soy agar with 5% sheep blood. The culture was then incubated until scant growth was visible, approximately 4 to 6 hours. Once growth was observed, the organisms were spotted in duplicate with 1 sample being overlayed with 1 *μ*L of 70% formic acid. After drying, 1 *μ*L of HCCA matrix is placed over the spot. Using the standard culture-based database, identifications were made via MALDI Biotyper^®^ Sirius CA system (US-IVD).

*Biotyper*^*^®^*^ *CA confidence scoring* the MALDI Biotyper^®^ Sirius CA system (US-IVD) produces confidence scores for each identification. The 3 categories for confidence scores are: no organism identification possible, low confidence identification, and high confidence identification. For the Sepsityper^®^ database, no organism identification is provided with a score of less than 1.6, low confidence identification is between 1.6 and 1.79, and high confidence identification is 1.8 or higher. For the culture-based database no organism identification score is less than 1.7, low confidence identification is between 1.7 and 1.99, and high confidence identification is a confidence score of 2.0 or greater.

*Time-to-results and hands-on time:* time-to-result was measured as the length of time from beginning the procedure to when the results were provided. Hands-on time was the approximate time during the procedure that requires processing by an individual. Both time-to-results and hands-on time were measured by timer for each processed sample and averaged for the MALDI-TOF MS methods. Since BioFire^®^ BCID2 was performed routinely in the clinical microbiology laboratory, time-to-results and hands-on time were estimated based on previously published reports [19]. The mean and standard error for the time-to-results and hands-on time was calculated and compared via 1-way analysis of variance (ANOVA).

*Statistical analysis:* bacterial and yeast identification from each diagnostic test were compared to standard culture-based identifications produced at the University of Maryland Medical Center clinical microbiology laboratory. For monomicrobial cultures, separate tables were created Gram-positive, Gram-negative, and yeast species. Within each group, positive identification rates were calculated for each species for Rapid Sepsityper^®^, Sepsityper^®^ extraction, total Sepsityper^®^, short-term culture, and Biofire^®^ BCID2. Total Sepsityper^®^ was defined as having at least 1 spot identified by either Rapid Sepsityper^®^ or Sepsityper^®^ extraction. Percent identified was calculated as proportion of samples with the correct species identification. For the MALDI- based diagnostics, Rapid Sepsityper^®^, Sepsityper^®^ extraction, total Sepsityper^®^, and short-term culture, percent identification was calculated for the proportion identified with at least low confidence, defined as having an identification with low or high confidence, and for the proportion calculate with only high confidence.

For polymicrobial cultures, total Sepsityper^®^, short-term culture, and BioFire^®^ FilmArray^®^ were compared. Polymicrobial cultures were defined as any blood culture containing 2 or more microorganisms. Complete identifications occurred when all organisms present and identified via standard culture methods were identified by the respective diagnostic test. A partial identification was defined as identifying at least 1 but not all microorganisms identified via traditional culture methods. All organisms identified during partial identifications were listed.

## 3. Results

A total of 301 prospective positive blood cultures from unique patients were collected during this study. Of the 273 monomicrobial blood cultures, 105 were Gram-positive bacteria, 157 were Gram-negative bacteria, and 11 were yeast species. The 28 remaining positive blood cultures were polymicrobial. Of the 28, 23 contained 2 organisms and 5 contained 3 organisms.

**Fig. 1.**
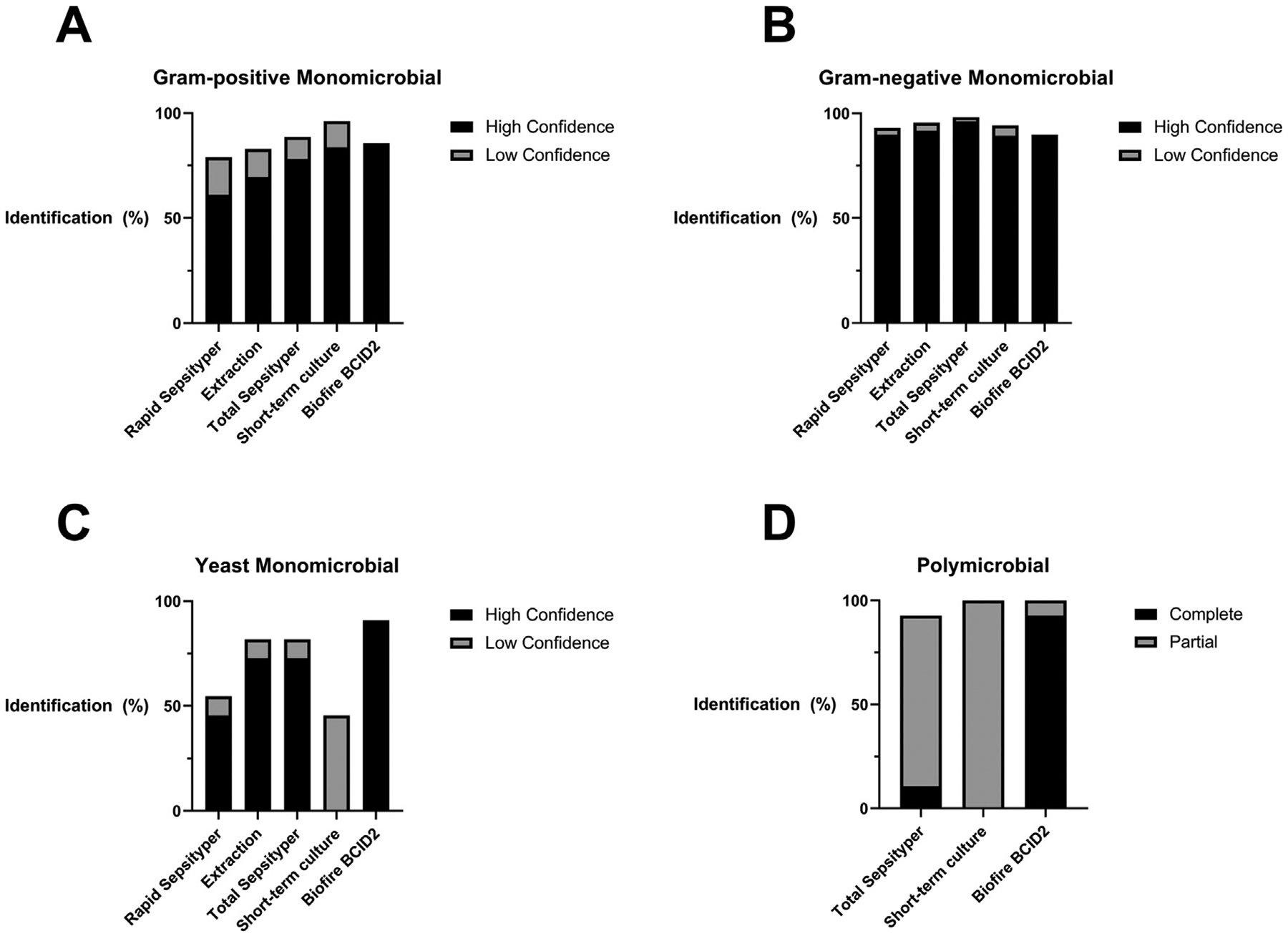
Shown are stacked bar graphs comparing percent identification of all compared diagnostic tests for monomicrobial cultures (A, B, C) and polymicrobial cultures (D). For monomicrobial cultures, (A) Gram-positive, (B) Gram-negative, and (C) yeast, the black is high confidence identifications, and the grey is low confidence. For (D) polymicrobial cultures, the black is complete identifications while the grey is partial identifications.

*Gram-positive monomicrobial blood cultures:* for monomicrobial positive blood cultures containing Gram-positive bacteria, Rapid Sepsityper^®^ identified 79.0% (83/105) with at least low confidence, identifications with either low or high confidence scores, and 61.0% (64/105) at high confidence (Supplementary Table 1). The discrepancy between low and high confidence identifications for Rapid Sepsityper^®^ was mostly attributed to lower confidence identification of *Staphylococcus* species. The Sepsityper^®^ extraction identified 82.9% (87/105) with at least low confidence and 69.5% (73/105) at high confidence (Fig. 1A). Like Rapid Sepsityper^®^, Sepsityper^®^ extraction discrepancy between low and high confidence identifications was attributed to *S. epidermidis* and *S. aureus*. In summation, total Sepsityper^®^ identified 88.6% (93/105) organisms with at least low confidence and 78.1% (82/105) at high confidence. Overall, Sepsityper^®^, both rapid and the extraction, demonstrated limited ability identifying *Streptococcus species*.

The short-term culture method was able to identify 96.2% (101/105) with at least low confidence and 83.8% (88/105) at high confidence. For short-term culture, the decrease in high confidence identifications was mostly caused by low confidence identifications of coagulase negative *Staphylococcus* (CoNS) excluding S. *epidermidis*. BioFire^®^ FilmArray^®^ BCID2 was able to identify 85.7% (90/105) of the organisms present. All 15 blood cultures that BioFire^®^ FilmArray^®^ BCID2 failed to identify contained organisms not included on the BCID2 panel.

*Gram-negative monomicrobial blood cultures:* of the 157 positive blood cultures collected containing Gram-negative bacteria, Rapid Sepsityper^®^ identified 93.0% (146/157) with at least low confidence and 89.8% (141/157) at high confidence (Supplementary Table 2). With at least low confidence, Sepsityper^®^ extraction identified 95.5% (150/157) of organisms, 91.7% (144/157) at high confidence (Fig. 1B). Total Sepsityper^®^ had 98.1% (154/157) percent identification with at least low confidence and 96.2% (151/157) at high confidence only. The short-term culture method identified 94.3% (148/157) of organisms with at least low confidence and 89.2% (140/157) at high confidence only. BioFire^®^ FilmArray^®^ BCID2 correctly identified 89.8% (141/157) of the Gram-negative organisms present. Of the 16 blood cultures that contained organisms BioFire^®^ FilmArray^®^ BCID2 failed to identify, 9 were not included in the BCID2 panel and 7 were correctly identified as Enterobacterales but no identified at the genus or species level. These 7 organisms were *Pantoea eucrina, Leclercia, Providencia rettgeri, Citrobacter koseri*, and *Citrobacter freundii* complex.

*Yeast monomicrobial blood cultures:* of the 11 positive blood cultures collected, all contain yeast species: 2 *Candida albicans*, 5 *Candida glabrata*, 3 *Candida parapsilosis*, and 1 *Candida lusitaniae*. Rapid Sepsityper^®^ identified with at least low confidence 54.5% (6/11) of yeast and 45.5% (5/11) at high confidence while Sepsityper^®^ extraction identified 81.8 (9/11) with at least low confidence and 72.7% (8/11) at high confidence (Supplementary Table 3). Combined, total Sepsityper^®^ identified 81.8 (9/11) with at least low confidence and 72.7% (8/11) at high confidence (Fig. 1C). The short-term culture method identified 45.5% (5/11) with low confidence scores and made no high confidence identifications. The low confidence and percent identification was due to the slow growing nature of the yeast species. Several of the specimens failed to have any growth appear within 6 hours. Finally, BioFire^®^ FilmArray^®^ BCID identified 90.9% (10/11) with the only failed identification from *Candida lusitaniae*, an organism not on the panel.

*Polymicrobial blood cultures:* identification of the 28 polymicrobial blood cultures by total Sepsityper^®^, short-term culture, and BioFire^®^ FilmArray^®^ BCID2 were compared. For total Sepsityper^®^, a complete identification, where all present organisms in culture were identified, was made for 10.7% (3/28) of blood cultures (Fig. 1D). Partial identifications, where at least 1 organism present was identified, occurred for 82.1% (23/28) of polymicrobial positive blood cultures (Supplementary Table 4). Total Sepsityper^®^ failed to identify any organisms for 7.1% (2/28) of polymicrobial blood cultures (Fig. D). Of the 5 blood cultures with 3 organisms, total Sepsityper^®^ was able to detect 2 organisms for 2 of the blood cultures.

The short-term culture method yielded only partial identifications for all 28 of the polymicrobial blood cultures. Furthermore, short-term culture was only able to identify 1 organism per blood culture whether there were 2 or 3 organisms identified by standard culture methods. BioFire^®^ FilmArray^®^ BCID2 identified all organisms present in 92.9% (26/28) of blood cultures and had partial identifications for the other 7.1% (2/28). For the 2 partial identifications, BioFire^®^ FilmArray^®^ BCID2 failed to identify *Pseudomonas* species not included on the BCID2 panel.

*Antimicrobial resistance identification: by BioFire*^*^®^*^ *FilmArray*^*^®^*^ *BCID2* Among the diagnostics that were compared, BioFire^®^ FilmArray^®^ BCID2 was the only platform capable of identifying antimicrobial resistance. Of the 301 blood cultures collected in this study, 55 had organisms with resistance genes identified. Among these were 13 with the CTX-M gene including 6 *K. pneumoniae*, 6 *E. coli*, and *1 Proteus species*. Additionally, there were 3 *K. pneumoniae* harboring a KPC, 25 *S. epidermidis* with a MecA gene detected, 11 *S. aureus* with a MecA and MREJ (MRSA) detected, and 3 *E. faecium* with a VanA/B gene. Despite being identified by BioFire^®^ FilmArray^®^ BCID2, phenotypic resistant organisms were not recovered in culture in 3 instances. This included 1 *K. pneumoniae* harboring a KPC, 1 S. aureus with a MecA and MREJ (MRSA), and 1 *E. faecium* with a VanA/C gene.

*Time-to-results and hands-on time:* the average time-to-results and hands-on time for the 301 blood cultures collected was calculated for each diagnostic test. Rapid Sepsityper^®^ yielded an average time-to-results of 35 (+/− 3.2) and hands-on time of 25 (+/− 1.4) minutes. Time-to-results and hands-on time for Sepsityper^®^ extraction were 52 (+/− 5.2) minutes and 40 (+/− 2.8) minutes, respectively. The short-term culture method required incubation time; thus time-to-results was much higher at 306 (+/− 29.3) minutes. Hands-on time for the short-term culture method was only 4 (+/−0.25) minutes. Finally, BioFire^®^ BCID2 yielded an approximate time-to-results of 65 (+/− 1.0) minutes and an estimated hands-on time of 5 minutes. Both average time-to-results and hands-on-time were significantly different by 1-way ANOVA (P < 0.001).

## 4. Discussion

Rapid and accurate identification of microorganisms directly from positive blood cultures is paramount in potentially improving antimicrobial therapy and patient outcomes during BSIs. Previously, all FDA-approved, direct from blood culture diagnostic methods were molecular-based assays with limited pathogen panels [20–23]. However, with the advent of MALDI-TOF MS in clinical microbiology laboratories as a high-throughput, cost effective diagnostic instrument with extensive pathogen panels, direct from blood culture assays for MALDI-TOF MS have been developed [24–26]. The first of these tests to FDA-cleared was the Bruker Sepsityper^®^. As novel MALDI-TOF MS methods for blood culture identification emerge, it is important to compare to existing molecular tests. Thus, this study compared MALDI-TOF MS platforms to a molecular-based, direct from blood culture assay, BioFire^®^ BCID2. This study compared accuracy of the Sepsityper^®^ kit (US-IVD), short-term culture, and BioFire^®^ BCID2 in parallel with monomicrobial cultures of Gram-positive and Gram-negative bacteria, and yeast and polymicrobial cultures. Moreover, time-to-results and hands-on time for each diagnostic test were measured and compared.

In this study, for monomicrobial cultures, Sepsityper^®^, including Rapid Sepsityper^®^ and total Sepsityper^®^ were consistent or better than previously published studies. Uniquely, this study performed Sepsityper^®^ extraction for every blood culture collected to evaluate its performance. In previous studies, Sepsityper^®^ extraction was only performed when instructed to by the approved Sepsityper^®^ protocol [16]. For Gram-positive monomicrobial cultures in our study, 61.0% (64/105) were correctly identified at high confidence by Rapid Sepsityper^®^ and 78.1% (82/105) were identified at high confidence by total Sepsityper^®^. Results from previous studies have demonstrated correct identification rates of monomicrobial Gram-positive blood cultures ranging from 60.0% to 70.0% for Rapid Sepsityper^®^ and 68.2% to 76.4% for total Sepsityper^®^ [27,28].

Like previous findings with direct from blood culture MALDI-TOF MS diagnostics, identification rates of Gram-negative monomicrobial cultures were higher than identification rates for Gram-positive monomicrobial cultures. For Rapid Sepsityper^®^ and total Sepsityper^®^, typical identification rates for Gram-negative blood cultures ranged from 78.9% to 82.2% and 78.9% to 91.0%, respectively [27–29]. Identification rates in our study for Gram-negative monomicrobial cultures was higher than previously published studies being 89.8% (141/157) and 96.2% (151/157) for Rapid Sepsityper^®^ and total Sepsityper^®^, respectively. The discrepancy between Gram-positive and Gram-negative identification rates has been theorized to be attributed to the bacterial cell wall; thus, use of formic acid is thought to be a potentially necessary component [30–32]. Another potential explanation for the lower Gram-positive identification rate is the known limitations of protein-based MALDI-TOF MS identifying coagulase-negative *Staphylococcus* and *Streptococus* at the species level [33–35]. While the identification of coagulase-negative *Staphylococcus* in our study is more successful than previously published, 69.4% (25/36) for Rapid Sepsityper^®^ and 91.7% (33/36) for total Sepsityper^®^, identification of *Streptococcus* remained problematic. The identification rates for Rapid Sepsityper^®^ and total Sepsityper^®^ of *Streptococcus* were 38.1% (8/21) and 61.9% (13/21), respectively.

Another MALDI-TOF MS blood culture identification method used in this study was the short-term culture method, sometimes known as the “short incubation” method in the literature. Previous studies have shown identification rates of monomicrobial cultures using this method of 65.3% to 87.9% for Gram-positives and 90.6% to 95.2% for Gram-negatives [36,37]. Consistently, the results from our study yielded identification rates of 83.8% and 89.2% for Gram-positives and Gram-negatives, respectively. As expected, given the short incubation time, the short-term culture method was unable to identify obligate anaerobes and other slow growing organisms. Interestingly, short-term culture did not struggle to identify *Streptococcus* with an identification rate of 95.2% (20/21). While the average estimated time-to-results for short-term culture were just over 5 hours, previous studies have demonstrated results from Gram-negative bacteria in as little as 2 to 5 hours [38].

BioFire^®^ BCID2 is a PCR-based molecular assay with 1 of the largest panels of molecular-based assays [39]. Data has shown that BioFire^®^ BCID2 has monomicrobial sensitivities as high as 98.6% for Gram-positives and 99.0% for Gram-negatives [40,41]. However, these studies either have high coverage rates ranging between 94.9% to 98.7%, many of the organisms present in blood culture are included on the panel, or organisms not on the panel are excluded from the study. In this study, cultures containing organisms not included on the panel were included as it best captures clinical performance. Moreover, the coverage rate of cultures collected in this study was 88.3% for all monomicrobial cultures; therefore, the identification rates of BioFire^®^ BCID2 in this study were lower than previous studies.

In addition to monomicrobial blood cultures, polymicrobial blood cultures were included in this study. MALDI-TOF MS is known to have limited utility for identifying polymicrobial cultures, especially directly from specimen [42]. For Sepsityper^®^, performance with polymicrobial cultures has been stated to yield poor results with at least 1 organism identification per polymicrobial culture being made under 65.0% of the time [43]. In this study, Sepsityper^®^ was able to identify at least 1 organism 92.9% (26/28) of the time with multiple correct identifications being made 17.9% of the time (5/28). The short-term culture method, as expected, was only able to identify single organisms since different colony morphologies are unable to be observed with short incubation [31]. Previously published results regarding performance of polymicrobial cultures for BioFire^®^ BCID2 widely vary having complete identification rates varying from less than 50.0% to 100% [40,41,44,45].

Along with accuracy, another critical component of rapid diagnos tic tests are the time-to-results. This study actively estimated processing time for each diagnostic test for each sample and averaged to compare, a major strength of this study. While Rapid Sepsityper^®^ and Sepsityper^®^ extraction have significantly shorter time-to-results (P < 0.001), hands-on time for these protocols is much longer. Furthermore, these times are consistent with what is reported currently in the literature [28,29]. The reduced time-to-results is important as it can potentially shorten time to effective therapy. However, the increased processing time may make Sepsityper^®^ more difficult to adopt in more burdened clinical microbiology laboratories. Therefore, batching of samples may be necessary to conserve time.

Another major factor when comparing diagnostic tests is cost. Cost especially important for laboratories without a lot of funds or resources. Although this study does not include a cost analysis, other published studies have estimated that cost of MALDI-TOF MS-based assay to be approximately 5 to 10 dollars per positive blood culture as opposed several hundred for molecular-based assays [28,29,46]. Moreover, as many clinical laboratories already MALDI-TOF MS present in their laboratories, reagent and instrument costs are reduced [47,48]. However, these estimated costs do not factor in additional costs of the Sepsityper^®^ kit and necessary software that is not present currently in many laboratories. Furthermore, the cost of the additional time required of a technologist to perform the different Sepsityper^®^ protocols must also be considered. Despite the potentially higher cost, 1 advantage of BioFire^®^ BCID2 over MALDI-TOF platforms such as Sepsityper^®^ and short-term culture is the ability to identify resistance. Currently, no MALDI-TOF MS platform is FDA-approved to identify antimicrobial resistance [49]. The ability for BioFire^®^ BCID2 to identify resistance genes has been demonstrated to potentially improve clinical outcomes [50]. While BioFire^®^ BCID2 is the only diagnostic platform that can identify resistance genes in this study, several other assays exist that were not evaluated. This includes other molecular assays such as Verigene^®^ blood culture panel (Luminex^®^ Corporation) and Genmark ePlex^®^ BCID (GenMarkDx^®^, Carlsbad, CA). Accelerate Pheno^®^ BC (Accelerate Diagnostics, Arizona) is an assay which determines minimum inhibitory concentrations (MICs) directly from positive blood culture. Future studies should be conducted to compare these platforms head-to-head [14].

This study was a comprehensive comparison that included many strengths including large sample size of clinical blood cultures, a diverse population of pathogens, and comparisons of time-to-results and hands-on time. Despite these many strengths, 1 potential limitation or bias of this study is that blood cultures were collected up to 12 hours after positivity. This delay could potentially affect the accuracy of some of the assays. However, no significant effect of delayed collection time within 12 hours after positivity and accuracy was observed in this study. Furthermore, collection of blood cultures closer to the time of positivity was less feasible for the research team. Overall, the several strengths of this study outweigh the potential bias introduced.

In conclusion, this thorough study evaluates several prominent diagnostic methods, Sepsityper^®^, short-term culture, and BioFire^®^ BCID2, that identify microorganisms directly from blood culture or with reduced time compared to standard identification methods. Each method is evaluated based on accuracy, time-to-results, and hands-on time using the same clinical blood cultures processed in parallel to reduce bias. Overall, each method has advantages and disadvantages; therefore, individual laboratory needs, and local epidemiology should be considered when implementing these tests.

## Supplementary Material

Supplemental 3

Supplemental 1

Supplemental 2

Supplemental 4

Supplementary material associated with this article can be found, in the online version, at doi:10.1016/j.diagmicrobio.2023.116018.
