# Supplementary material for: Comparison of 3 diagnostic platforms for identification of bacteria and yeast from positive blood culture bottles
Source: Diagn Microbiol Infect Dis. Author manuscript; Available in PMC 2026 Jul 21. (PMC13387351; doi:10.1016/j.diagmicrobio.2023.116018)
Supplement: Supplemental 3 [file NIHMS2192660-supplement-Supplemental_3.docx]

| % |  | 100 | 100 | 100 | 0 | 90.9 |
| --- | --- | --- | --- | --- | --- | --- |
| Biofire |  | 2 | 5 | 3 | 0 | 10 |
| Scum | % | 0 | 0 | 0 | 0 | 0 |
|  | # high confidence | 0 | 0 | 0 | 0 | 0 |
|  | % | 100 | 0 | 100 | 0 | 45.4 |
|  | # low confidence | 2 | 0 | 3 | 0 | 5 |
| Total Sepsityper | % | 100 | 60 | 100 | 0 | 73 |
|  | # high confidence | 2 | 3 | 3 | 0 | 8 |
|  | % | 100 | 60 | 100 | 100 | 81.8 |
|  | # Low confidence | 2 | 3 | 3 | 1 | 9 |
| Extraction | % | 100 | 60 | 100 | 0 | 72.7 |
|  | # high confidence | 2 | 3 | 3 | 0 | 8 |
|  | % | 100 | 60 | 100 | 100 | 81.8 |
|  | # low confidence | 2 | 3 | 3 | 1 | 9 |
| Rapid Sepsityper | % | 100 | 20 | 33.3 | 100 | 45.4 |
|  | # high confidence | 2 | 1 | 1 | 1 | 5 |
|  | % | 100 | 20 | 67.7 | 100 | 54.5 |
|  | # low confidence obetter | 2 | 1 | 2 | 1 | 6 |
| N | | 2 | 5 | 3 | 1 | 11 |
| Species |  | *Candida albicans* | *Candida glabrata* | *Candida parapsilosis* | *Candida lusitaniae* | Total |

**Table 3. The 11 yeast monomicrobial blood cultures and the percent identification for each platform.**
