# Supplementary material for: Comparison of 3 diagnostic platforms for identification of bacteria and yeast from positive blood culture bottles
Source: Diagn Microbiol Infect Dis. Author manuscript; Available in PMC 2026 Jul 21. (PMC13387351; doi:10.1016/j.diagmicrobio.2023.116018)
Supplement: Supplemental 1 [file NIHMS2192660-supplement-Supplemental_1.docx]

| Biofire | % identified | 100.0 | 100.0 | 100.0 | 100.0 | 100.0 | 100.0 | 0 | 0 | 0 | 0 | 0 | 0 | 85.7 |
| --- | --- | --- | --- | --- | --- | --- | --- | --- | --- | --- | --- | --- | --- | --- |
|  |  | 24 | 12 | 23 | 6 | 4 | 21 | 0 | 0 | 0 | 0 | 0 | 0 | 90 |
| Scum | % identified | 87.5 | 58.3 | 95.7 | 100.0 | 100.0 | 95.2 | 0.0 | 60.0 | 0.0 | 66.7 | 66.7 | 50.0 | 83.8 |
|  | # high confidence | 21 | 7 | 22 | 6 | 4 | 20 | 0 | 3 | 0 | 2 | 2 | 1 | 88 |
|  | % identified | 95.8 | 100.0 | 100.0 | 100.0 | 100.0 | 100.0 | 0.0 | 80.0 | 100.0 | 100.0 | 66.7 | 100.0 | 96.2 |
|  | # low confidence better | 23 | 12 | 23 | 6 | 4 | 21 | 0 | 4 | 1 | 3 | 2 | 2 | 101 |
| Total Sepsityper | % | 88 | 100 | 91 | 83 | 75 | 62 | 0 | 40 | 0 | 100 | 33 | 50 | 78 |
|  | # high confidence | 21 | 12 | 21 | 5 | 3 | 13 | 0 | 2 | 0 | 3 | 1 | 1 | 82 |
|  | % identified | 95.8 | 100.0 | 100.0 | 100.0 | 75.0 | 76.2 | 0.0 | 60.0 | 100.0 | 100.0 | 66.7 | 50.0 | 88.6 |
|  | # low confidence or better | 23 | 12 | 23 | 6 | 3 | 16 | 0 | 3 | 1 | 3 | 2 | 1 | 93 |
| Extracation | % identified | 79 | 100.0 | 83 | 67 | 25 | 52 | 0 | 40 | 0 | 100 | 33 | 50 | 70 |
|  | # high confidence | 19 | 12 | 19 | 4 | 1 | 11 | 0 | 2 | 0 | 3 | 1 | 1 | 73 |
|  | % identified | 91.7 | 100.0 | 100.0 | 100.0 | 25.0 | 66.7 | 0.0 | 40.0 | 100.0 | 100.0 | 66.7 | 50.0 | 82.9 |
|  | # low confidence obetter | 22 | 12 | 23 | 6 | 1 | 14 | 0 | 2 | 1 | 3 | 2 | 1 | 87 |
| Rapid Sepsityper | % identified | 67.7 | 75.0 | 78.3 | 83.3 | 75.0 | 38 | 0 | 40 | 0 | 67.7 | 33.3 | 0 | 61 |
|  | # Identified high confidence | 16 | 9 | 18 | 5 | 3 | 8 | 0 | 2 | 0 | 2 | 1 | 0 | 64 |
|  | % identiied | 91.7 | 100.0 | 91.3 | 83.3 | 75.0 | 57.1 | 0.0 | 60.0 | 0.0 | 66.7 | 66.7 | 50.0 | 79.0 |
|  | # low confidence | 22 | 12 | 21 | 5 | 3 | 12 | 0 | 3 | 0 | 2 | 2 | 1 | 83 |
| N |  | 24 | 12 | 23 | 6 | 4 | 21 | 1 | 5 | 1 | 3 | 3 | 2 | 105 |
| Species | | *S. epidermidis* | *Other CoNS* | *S. aureus* | *E. faecalis* | *E. faecium* | *Streptococcus spp.* | *Cutibacterium acnes* | *Micrococcus* | *Pediococcus* | *Rothia* | *Corynebacterium* | *Bacillus species* | Total |

**Table 1. All monomicrobial blood cultures containing Gram-positive bacteria and the percent identification for each compared diagnostic test.**
