# Supplementary material for: Comparison of 3 diagnostic platforms for identification of bacteria and yeast from positive blood culture bottles
Source: Diagn Microbiol Infect Dis. Author manuscript; Available in PMC 2026 Jul 21. (PMC13387351; doi:10.1016/j.diagmicrobio.2023.116018)
Supplement: Supplemental 2 [file NIHMS2192660-supplement-Supplemental_2.docx]

| % | | 100.0 | 100.0 | 100.0 | 100.0 | 100.0 | 100.0 | 100.0 | 0.0 | 0.0 | 0.0 | 0.0 | 0.0 | 0.0 | 0.0 | 100.0 | 0.0 | 0.0 | 0.0 | 100.0 | 0.0 | 100.0 | 0.0 | 0.0 | 100.0 | 89.8 |
| --- | --- | --- | --- | --- | --- | --- | --- | --- | --- | --- | --- | --- | --- | --- | --- | --- | --- | --- | --- | --- | --- | --- | --- | --- | --- | --- |
| Biofire | | 44 | 35 | 6 | 4 | 15 | 4 | 17 | 0 | 0 | 0 | 0 | 0 | 0 | 0 | 2 | 0 | 0 | 0 | 2 | 0 | 1 | 0 | 0 | 11 | 141 |
| Scum | % identified | 100.0 | 100.0 | 83.3 | 100.0 | 100.0 | 100.0 | 100.0 | 100.0 | 0.0 | 100.0 | 100.0 | 100.0 | 100.0 | 100.0 | 0.0 | 0.0 | 100.0 | 0.0 | 0.0 | 100.0 | 100.0 | 100.0 | 0.0 | 100.0 | 94.3 |
|  | # high confidence | 42 | 34 | 5 | 4 | 13 | 4 | 16 | 1 | 0 | 0 | 1 | 3 | 1 | 1 | 0 | 0 | 1 | 0 | 0 | 1 | 1 | 1 | 0 | 11 | 140 |
|  | % identified | 95.5 | 97.1 | 83.3 | 100.0 | 86.7 | 100.0 | 94.1 | 50.0 | 0.0 | 0.0 | 100.0 | 100.0 | 100.0 | 100.0 | 0.0 | 0.0 | 100.0 | 0.0 | 0.0 | 100.0 | 100.0 | 100.0 | 0.0 | 100.0 | 89.2 |
|  | # low confidence obetter | 44 | 35 | 5 | 4 | 15 | 4 | 17 | 2 | 0 | 1 | 1 | 3 | 1 | 1 | 0 | 0 | 1 | 0 | 0 | 1 | 1 | 1 | 0 | 11 | 148 |
| Total Sepsityper | % identified | 97.7 | 100.0 | 100.0 | 100.0 | 93.3 | 100.0 | 94.1 | 100.0 | 0.0 | 0.0 | 100.0 | 100.0 | 100.0 | 100.0 | 100.0 | 100.0 | 100.0 | 100.0 | 50.0 | 100.0 | 100.0 | 100.0 | 100.0 | 100.0 | 96.2 |
|  | # high confidence | 43 | 35 | 6 | 4 | 14 | 4 | 16 | 2 | 0 | 0 | 1 | 3 | 1 | 1 | 2 | 1 | 1 | 1 | 1 | 1 | 1 | 1 | 1 | 11 | 151 |
|  | % identified | 100.0 | 100.0 | 100.0 | 100.0 | 100.0 | 100.0 | 94.1 | 100.0 | 0.0 | 100.0 | 100.0 | 100.0 | 100.0 | 100.0 | 100.0 | 100.0 | 100.0 | 100.0 | 50.0 | 100.0 | 100.0 | 100.0 | 100.0 | 100.0 | 98.1 |
|  | # low confidence or better | 44 | 35 | 6 | 4 | 15 | 4 | 16 | 2 | 0 | 1 | 1 | 3 | 1 | 1 | 2 | 1 | 1 | 1 | 1 | 1 | 1 | 1 | 1 | 11 | 154 |
| Extraction | % identified | 97.7 | 91.4 | 83.3 | 100.0 | 86.7 | 100.0 | 88.2 | 100.0 | 0.0 | 0.0 | 100.0 | 100.0 | 100.0 | 100.0 | 100.0 | 100.0 | 100.0 | 100.0 | 0.0 | 100.0 | 100.0 | 100.0 | 100.0 | 100.0 | 91.7 |
|  | # high confidence | 43.0 | 32 | 5 | 4 | 13 | 4 | 15 | 2 | 0 | 0 | 1 | 3 | 1 | 1 | 2 | 1 | 1 | 1 | 0 | 1 | 1 |  | 1 | 11 | 144 |
|  | % identified | 100.0 | 94.3 | 100.0 | 100.0 | 93.3 | 100.0 | 94.1 | 100.0 | 0.0 | 100.0 | 100.0 | 100.0 | 100.0 | 100.0 | 100.0 | 100.0 | 100.0 | 100.0 | 0.0 | 100.0 | 100.0 | 100.0 | 100.0 | 100.0 | 95.5 |
|  | # low confidence or better | 44 | 33 | 6 | 4 | 14 | 4 | 16 | 2 | 0 | 1 | 1 | 3 | 1 | 1 | 2 | 1 | 1 | 1 | 0 | 1 | 1 | 1 | 1 | 11 | 150 |
| Rapid Sepsityper | % identified | 95.5 | 91.4 | 100.0 | 100.0 | 86.7 | 100.0 | 94.1 | 100.0 | 0.0 | 0.0 | 100.0 | 66.7 | 100.0 | 0.0 | 50.0 | 100.0 | 100.0 | 0.0 | 50.0 | 100.0 | 100.0 | 100.0 | 0.0 | 100.0 | 89.8 |
|  | # high confidence | 42 | 32 | 6 | 4 | 13 | 4 | 16 | 2 | 0 | 0 | 1 | 2 | 1 | 0 | 1 | 1 | 1 | 0 | 1 | 1 | 1 | 1 | 0 | 11 | 141 |
|  | % identified | 97.7 | 94.3 | 100.0 | 100.0 | 93.3 | 100.0 | 94.1 | 100.0 | 0.0 | 0.0 | 100.0 | 66.7 | 100.0 | 0.0 | 100.0 | 100.0 | 100.0 | 100.0 | 50.0 | 100.0 | 100.0 | 100.0 | 0.0 | 100.0 | 93.0 |
|  | # low confidence better | 43 | 33 | 6 | 4 | 14 | 4 | 16 | 2 | 0 | 0 | 1 | 2 | 1 | 0 | 2 | 1 | 1 | 1 | 1 | 1 | 1 | 1 | 0 | 11 | 146 |
| N | | 44 | 35 | 6 | 4 | 15 | 4 | 17 | 2 | 1 | 1 | 1 | 3 | 1 | 1 | 2 | 1 | 1 | 1 | 2 | 1 | 1 | 1 | 1 | 11 | 157 |
| Species | | *Escherichia coli* | *Klebsiella pneumoniae* | *Klebsiella oxytoca* | *Klebsiella aerogenes* | *Enterobacter cloacae* complex | *Proteus* species | *Pseudomonas aeruginosa* | *Pseudomonas putida* | *Moraxella* | *Pantoea* | *Citrobacter koseri* | *Citrobacter freundii* complex | *Providencia rettgeri* | *Bacteroides theatiomicron* | *Bacteroides fragilis* | *Capnocytophaga* | *Delftia* | *Veillonella species* | *Haemophilus influenzae* | *Pasteurella multocida* | *Stenotrophomonas maltophila* | *Leclercia* | *Tissierella* | *Serratia marcescens* | Total |

**Table 2. All monomicrobial blood cultures containing Gram-negative bacteria and the percent identification for each compared diagnostic test.**
