# Supplementary material for: Comparison of 3 diagnostic platforms for identification of bacteria and yeast from positive blood culture bottles
Source: Diagn Microbiol Infect Dis. Author manuscript; Available in PMC 2026 Jul 21. (PMC13387351; doi:10.1016/j.diagmicrobio.2023.116018)
Supplement: Supplemental 4 [file NIHMS2192660-supplement-Supplemental_4.docx]

| BioFire | Partial IDs |  |  |  |  |  |  |  |  |  |  |  |  |  |  | *S. epidermidis* |  |  |  |  |  |  |  |  | *S. marcescens, S. maltophila* |  |  |
| --- | --- | --- | --- | --- | --- | --- | --- | --- | --- | --- | --- | --- | --- | --- | --- | --- | --- | --- | --- | --- | --- | --- | --- | --- | --- | --- | --- |
|  | % completely identified |  | 100 | 100 | 100 | 100 | 100 | 100 | 100 | 100 | 100 | 100 | 100 | 100 | 100 | 0 | 100 | 100 | 100 |  |  | 100 | 100 | 100 | 0 | 100 | 92.9 |
|  | # Complete IDs |  | 2 | 1 | 1 | 1 | 1 | 2 | 2 | 1 | 1 | 2 | 2 | 2 | 1 | 0 | 1 | 1 | 1 |  |  | 1 | 1 | 1 | 0 | 1 | 26.0 |
|  | % partially identified |  | 0 | 0 | 0 | 0 | 0 | 0 | 0 | 0 | 0 | 0 | 0 | 0 | 0 | 100 | 0 | 0 | 0 |  |  | 0 | 0 | 0 | 100 | 0 | 7.1 |
|  | # Partial IDs |  | 0 | 0 | 0 | 0 | 0 | 0 | 0 | 0 | 0 | 0 | 0 | 0 | 0 | 1 | 0 | 0 | 0 |  |  | 0 | 0 | 0 | 1 | 0 | 2 |
| Scum | Partial IDs |  | *2 E. coli* | *E. coli* | *E. coli* | *E. cloacae complex* | *P. aeruginosa* | *2 E. faecalis* | *1 K. pneumoniae, 1 S. epidermidis* | *K. pneumoniae* | *E. cloacae complex* | *2 S. aureus* | *1 S. aureus, 1 S. epidermidis* | *Proteus species* | *CoNS* | *S. epidermidis* | *S. agalactiae* | *S. marcescens* | *P. aeruginosa* |  |  | *K. pneumoniae* | *P. aeruginosa* | *S. aureus* | *S. maltophila* | *K. aerogenes* |  |
|  | % completely identified |  | 0 | 0 | 0 | 0 | 0 | 0 | 0 | 0 | 0 | 0 | 0 | 0 | 0 | 0 | 0 | 0 | 0 |  |  | 0 | 0 | 0 | 0 | 0 | 0.0 |
|  | # Complete IDs |  | 0 | 0 | 0 | 0 | 0 | 0 | 0 | 0 | 0 | 0 | 0 | 0 | 0 | 0 | 0 | 0 | 0 |  |  | 0 | 0 | 0 | 0 | 0 | 0.0 |
|  | % partially identified |  | 100 | 100 | 100 | 100 | 100 | 100 | 100 | 100 | 100 | 100 | 100 | 100 | 100 | 100 | 100 | 100 | 100 |  |  | 100 | 100 | 100 | 100 | 100 | 100.0 |
|  | # Partial IDs |  | 2 | 1 | 1 | 1 | 1 | 2 | 2 | 1 | 1 | 2 | 2 | 2 | 1 | 1 | 1 | 1 | 1 |  |  | 1 | 1 | 1 | 1 | 1 | 28.0 |
| Total Sepsityper | Partial IDs |  | *2 E. coli* | *E. coli* |  | *E. cloacae complex* | *E. faecalis* | *No ID, E. faecalis* | *K. pneumoniae, No ID* |  |  | *1 S. aureus, 1 S. pyogenes* | *1 S. aureus, 1 S. epidermidis* | *2 Proteus species* | *CoNS* | *S. epidermidis* | *S. agalactiae* | *S. marcescens* | *P. aeruginosa* |  |  | *K. pneumoniae* | *S. parasanguis, P. aeruginosa* | *S. agalactiae* | *S. maltophila* | *K. aerogenes, E. faecalis* |  |
|  | % completelidentified |  | 0 | 0 | 100 | 0 | 0 | 0 | 0 | 100 | 100 | 0 | 0 | 0 | 0 | 0 | 0 | 0 | 0 |  |  | 0 | 0 | 0 | 0 | 0 | 10.7 |
|  | # Complete IDs |  | 0 | 0 | 1 | 0 | 0 | 0 | 0 | 1 | 1 | 0 | 0 | 0 | 0 | 0 | 0 | 0 | 0 |  |  | 0 | 0 | 0 | 0 | 0 | 3.0 |
|  | % |  | 100 | 100 | 0 | 100 | 100 | 50 | 50 | 0 | 0 | 100 | 100 | 100 | 100 | 100 | 100 | 100 | 100 |  |  | 100 | 100 | 100 | 100 | 100 | 82.1 |
|  | # Partial IDs |  | 2 | 1 | 0 | 1 | 1 | 1 | 1 | 0 | 0 | 2 | 2 | 2 | 1 | 1 | 1 | 1 | 1 |  |  | 1 | 1 | 1 | 1 | 1 | 23.0 |
| N | |  | 2 | 1 | 1 | 1 | 1 | 2 | 2 | 1 | 1 | 2 | 2 | 2 | 1 | 1 | 1 | 1 | 1 |  |  | 1 | 1 | 1 | 1 | 1 | 28.0 |
| Organisms Identified | | **Two organisms** | *E. coli, K. pneumoniae* | *E. coli, K. oxytoca* | *E. coli, S. agalactiae* | *E. faecalis, E. cloacae complex* | *E. faecalis, P. aeruginosa* | *E. faecalis, S. epidermidis* | *K. pneumoniae, S. epidermidis* | *K. pneumoniae, Proteus species* | *S. aureus, E. cloacae complex* | *S. aureus, S. pyogenes* | *S. aureus, S. epidermidis* | *CoNS, Proteus species* | *CoNS, E. faecalis* | *P. stutzeri, S. epidermidis* | *S. epidermidis, S. agalactiae* | *S. epidermidis, S. marcescens* | *P. aeruginosa, Strep species* |  | **Three organisms** | *E. faecalis, E. cloacae complex, K. pneumoniae* | *S. epidermidis, Strep species, P. aeruginosa* | *E. faecalis, S. aureus, S. agalactiae* | *S. marcescens, S. maltophila, P. putida* | *K. aerogenes, E. faecalis, E. cloacae complex* | Total |

**Table 4. Comparison between Total Sepsityper®, Scum, and FilmArray® to identify polymicrobial blood cultures. Percent of partial identifications and complete identifications are presented.**
